# Evaluating differently tutored groups in problem-based learning in a German dental curriculum: a mixed methods study

**DOI:** 10.1186/s12909-015-0505-0

**Published:** 2016-01-14

**Authors:** Susanne Gerhardt-Szep, Florian Kunkel, Andreas Moeltner, Miriam Hansen, Anja Böckers, Stefan Rüttermann, Falk Ochsendorf

**Affiliations:** Department of Operative Dentistry, Center for Dentistry and Oral Medicine (Carolinum), Medical Faculty, Goethe University of Frankfurt am Main, Theodor-Stern-Kai 7, Building 29, 60596 Frankfurt am Main, Germany; Private Practice, Bergerstraße 159, 60385 Frankfurt am Main, Germany; Competence Center for Assessment in Medicine, Medical Faculty, University of Heidelberg, Im Neuenheimer Feld 346, 69120 Heidelberg, Germany; Institute of Psychology, Interdisziplinäres Kolleg Hochschuldidaktik (IKH), Goethe University of Frankfurt am Main, Senckenberganlage 15, 60325 Frankfurt am Main, Germany; Medical Faculty, Institute of Anatomy and Cell Biology, University in Ulm, Albert-Einstein Allee 11, 89081 Ulm, Germany; Medical Faculty, Institute of Dermatology, Goethe University of Frankfurt am Main, Theodor-Stern-Kai 7, Building 28, 60596 Frankfurt am Main, Germany

**Keywords:** Assessment, Clinical tutor, Curriculum, Effectiveness, Evaluation, Faculty development, Focus groups, Knowledge, Mixed methods research, Problem-based learning

## Abstract

**Background:**

It is still unclear to what extent the PBL tutor affects learning in PBL-sessions. This mixed-methods study (Part 1 and 2) evaluated the effects of facilitative (f) versus non-facilitative (nf) tutoring roles on knowledge-gain and group functioning in the field of endodontics.

**Methods:**

Part 1 was a quantitative assessment of tutor effectiveness within a prospective, experimental, single-blind, stratified, randomized, two-group intervention study. Participants attended PBL in the context of a hybrid curriculum. A validated questionnaire was used and knowledge assessments were conducted before and after the intervention. External observers rated tutor performance. Part 2 was a qualitative assessment of tutor effectiveness and consisted of semi-structured expert interviews with tutors and focus group discussions with students.

**Results:**

Part 1: f tutors obtained significantly higher scores than nf tutors with respect to learning motivation and tutor effectiveness (*p* ≤ 0.05). nf tuition resulted in a slightly larger knowledge gain (*p* = 0.08). External observers documented a significantly higher activity among facilitative tutors compared to non-facilitative tutors.

Part 2: Tutors found the f role easier although this led to a less autonomous working climate. The students rated f tutoring as positive in all cases.

**Conclusions:**

With respect to PBL-group performance, students felt that groups guided in a non-facilitative fashion exhibited a higher level of independence and autonomy, especially with increasing PBL experience. In addition, students reported that more preparation was necessary for sessions guided by a non-facilitative tutor. Tutors were able to modify their role and influence group processes in a controlled manner. Results are useful for future “Train-the-Teacher” sessions.

## Background

In their 2012 guidelines entitled “Recommendations on higher education qualifications for the healthcare system” for the advancement of dental medicine at German universities, the German Council of Science and Humanities endorses the problem-oriented and patient-centred learning as a great step forward [1].

Since the introduction of problem-based Learning (PBL) in the 1960s, many empirical studies employing various evaluation methods have evaluated the effectiveness of PBL [2–17]. Most authors chose a qualitative approach using interviews of various types [18–20]. Only approximately 10 % of these studies employed the mixed-methods approach, although such studies are particularly informative because they address the research question on various methodological levels [21–24]. Literature suggests that it can be used as an effective approach, but this is not always the case [25]. Actually it is known, that PBL aims to improve the hypothetico-deductive reasoning (HDR) of students. However, HDR brings with it a high cognitive load, so the full use of PBL is not advocated for medical curricula [25]. In the literature, there is general agreement on the definition of PBL: small groups of students work interactively on the solution of problems while being supported by a tutor. Thereby, distinction is made between sessions necessitating the student’s presence in the group and periods of individual learning. The tutor is required to accompany, rather than control, the learning process. The role of the tutor may be described as conducive or facilitative [26]. Facilitation requires understanding of the learning process, primarily involves monitoring of student learning and promotion of effective group function [27]. In order to effect student learning in the small group PBL session, the facilitator must be informed about and be acutely aware of his/her role [28]. Such performance was aptly described by Hamdy as follows: “The key is when, how and how much guidance should be provided” [29]. Nonetheless, clear description of the extent of guidance and precise delineation of tutor performance are rare in the literature as are reports of controlled, clinical studies of PBL. Dolmans et al. [30] alerted to this gap and interpreted it as a guiding principle for future research. However few studies aiming to elucidate open questions on the tutor’s role have been published since this paper.

The present prospective controlled study attempted to evaluate precisely defined tutoring styles. To account for the complexity of the topic, we chose a mixed-methods study design. Part 1 (quantitative part) addressed the following questions:

RQ1: How do the different tutoring styles (facilitative and non-facilitative) affect learning motivation, tutor effectiveness, group dynamics, and learning success in PBL sessions for dental students attending a hybrid curriculum?

RQ2: Can external observers distinguish between the different tutoring styles, based on the tutors‘activities performed?

Part 2 of the study (qualitative part) attempted to answer the following question:

RQ3: How do students and tutors rate the different tutoring styles in the PBL sessions?

## Methods

### Sample-size calculation

Sample-size calculation was based on an intermediate effect size (d = 0.5). For a one-sided test (i.e., assuming that the effects of facilitative tutoring are not equal to those of non-facilitative tutoring) at a power of 0.8, the sample size per study group amounted to *n* = 50.

### Study population and exclusion criteria

Overall, 4 PBL tutors and 106 students (41 men, 65 women; mean age = 25.2 years) participated in the study. The students were attending the first clinical semester (sixth subject-specific semester) of the dental school during summer term 2008, winter term 2008, or summer term 2009. Table 1 shows the distribution of the study population, stratified by age and gender. Inclusion criteria for the tutors were several years of experience with PBL tutorials and a complete dental training including the license to practice. All participating students had to be attending the sixth subject-specific semester and were not allowed to have any experience with PBL tutorials or to have attended any lectures on the subject of endodontology at study start. Due to previously defined exclusion criteria, a total of *n* = 5 were excluded from the final study design, so that the study population consisted of *n* = 101. Prior to study start, the study protocol was approved by the local Ethics Committee (164/08).

**Table 1:** Distribution of study population stratified for age and gender

Semester	Facilitative			Non-facilitative		
	Men	Women	Mean age (years)	Men	Women	Mean age (years)
SS 2008	4	8	26.50	4	8	25.41
WS 2008	6	9	25.00	5	11	25.73
SS 2009	10	13	24.52	10	13	24.91

### Dentistry curriculum

The Dental School at the University of Frankfurt am Main, Germany employs a hybrid curricular structure that combines conventional (i.e., lectures, courses, seminars) and modern (i.e., eLearning, PBL, blended learning) teaching methods. None of the participating students had previously worked with PBL, while all tutors had several years of PBL experience and were established experts (i.e., content expert tutors) in both theoretical and practical endodontics. This module focused on endodontics is curriculary integrated and part of the first clinical semester.

### Concept of PBL

The PBL module consisted of 7 steps, in accordance with the procedure used at the University of Maastricht [31]. Steps 1 to 5 (1. Clarification of terms and concepts; 2. Formulation of problem statement; 3. Brainstorm; 4. Categorizing and structuring of brainstorm; 5. Formulation of learning objectives) were dealt with during Session 1, while Step 6 (Self-study) consisted of the individual processing of learning objectives. Step 7 (Post-discussion and reflection on learning process) took place in Session 2 scheduled 1 week after Session 1. Each PBL session lasted 90 min, group work in connection with each PBL case lasted 180 min. A total number of eight cases were included into this study.

### Instruction of students

Prior to the planned PBL module entitled “Endodontics”, the students received verbal instruction lasting approximately 60 min, focusing on the following issues: What does PBL stand for? Definition of steps, timeframe, tips for literature search, allocation of roles in the group (time keeper; summarist; moderator; writer), and the role of the tutor. In addition, a video film about PBL was shown, and a handout was provided (32).

### Training of tutors

All tutors (*n* = 4; 3 women, 1 man) underwent a “Train-the-Teacher” seminar (60 min) to refresh their PBL experience and to familiarize themselves with the facilitative (f) and non-facilitative (nf) tutoring styles (Table 2). The tutoring styles were defined in accordance with publications by Walsh [32] and Tuckman [33] as well as observations made on occasion of a PBL-guest visit at Charité, University of Berlin. At the end of the “Train-the-Teacher” seminar it was checked, if the tutors can correctly act in the different styles using video-analysis. The PBL module and the “Train-the-Teacher” seminar were supervised by an established expert (Master of Medical Education) in this field.

**Table 2:** Characteristics of facilitative and non-facilitative tutoring (as defined in the study protocol. Group interaction phases are F = forming, S = storming, and N = norming)

Facilitative tutoring (f)	Non-facilitative tutoring (nf)
Tutor	Tutor
1. offers orientation and explanation (F).	1. is participative and delegates (F).
2. is aware of defined learning objectives (S).	2. is not aware of defined learning objectives (S).
3. intervenes actively in intra-group processes, if required (N).	3. intervenes in acute necessity in intra-group processes (N).
4. helps the group in the “forming” process (F).	4. doesn’t help the group in the forming process (F).
5. recognize and specify arising conflicts (S).	5. recognize, but doesn’t specify arising conflicts (S).
6. encourages participation of members, if necessary (S).	6. doesn’t encourages participation of members (S).
7. facilitates actively group collaboration (S).	7. does’t facilitates actively group collaboration (S).
8. offers during the session corrective feedback, if necessary (N).	8. doesn’t offer during the session corrective feedback (N).

## Study procedures

### Quantitative part (Part 1)

Part 1 was a prospective, experimental, single-blind, stratified, randomized, two-group intervention study comparing a number of variables before and after the PBL module using knowledge assessments. The students were stratified for age and gender using the software RandList 1.2 (DatInf GmbH, Tübingen, Germany). They were randomly allocated to one of the two groups. Randomization codes were generated locally and were treated confidentially before study start. The study participants were not informed of the planned interventions (single-blind study design).

### PBL cases

**Fig. 1:**
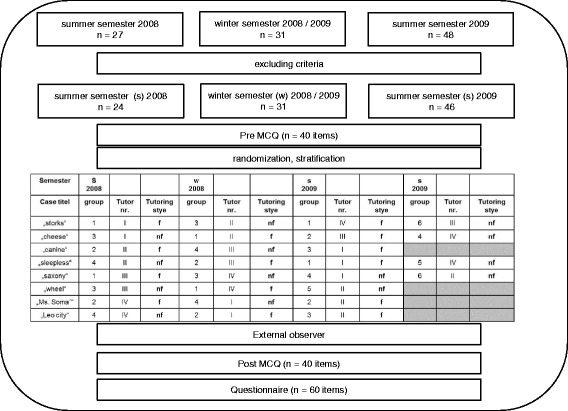
Flowchart of the study (nf = non-facilitative, f = facilitative)

In each semester students were allocated. Each group worked on two different PBL cases. Not all students worked on each case (Fig. 1). However, the contents developed by the group were presented to all students of the semester in a plenary lecture session at the end of each PBL case study, ensuring that all participants were informed. Thus, all participants were able to complete the post-PBL test.

PBL was scheduled on 2 days (Tuesday and Thursday) each week with different groups. One week intervened between the two sessions of each group (for example from Tuesday to Tuesday or from Thursday to Thursday). The weekly timetable of the hybrid dental curriculum included few PBL and more plenary lecture sessions. On the respective PBL days (Tuesday and Thursday), two groups were taught in the same location/class room (sufficiently far away from each other). To prevent any detection of the differences in the tutoring style we ensured that both groups were either accompanied in the same style in this joint session. Groups were tutored by just one style (facilitative or non-facilitative) that was determined before the start of the study. However, the tutors acted in both a facilitative or non-facilitative fashion, depending on the group. The group members were unaware to which one of the two tutoring styles they had been allocated. Each PBL case consisted of eight learning objectives that were known to the tutors only. Thus, a total of 64 learning objectives were elaborated for the eight PBL cases.

### External observers

Each PBL group received an external observer who confirmed the allocated tutoring role and its proper application by the tutor. Before study start, the external observer was trained to assess the frequency of activities initiated by the tutor or the group.

### Pre- and post-PBL tests

Both groups underwent a written test (multiple-choice questionnaire) before and after the PBL module to assess the participants’ level of knowledge. Neither test was announced. Both tests consisted of 40 multiple-choice questions that were different in the pre- and post-PBL tests. The tests were to be completed within 20 min in an anonymized fashion, ensuring however that the pre- and post-PBL tests could be matched for each participant. The contents of the tests remained unchanged during the study period (three semesters). Values of Cronbach‘s alpha amounted to 0.63 and 0.67 for the pre- and post-PBL tests, while the values for the difficulty of the pre- and post-PBL tests were 0.40 and 0.53, respectively.

### Study questionnaire

Effectiveness of the tutoring styles was assessed on the basis of a questionnaire completed by the students. The contents of the questionnaire (*n* = 60 questions) were based on studies by Visschers-Pleijers et al. [34] and Dolmans & Ginns [35] and matched the PBL questionnaire used at Charité, Berlin [36]. The questionnaire consisted of three sections (i.e., group interaction, tutor effectiveness, general comments on PBL), with a section for additional remarks provided (Table 3). Questions were answered using a Likert scale ranging from one to five (1 = completely disagree, 2 = disagree, 3 = neutral, 4 = agree, 5 = completely agree). In addition, an observation chart (“manipulation check“) was constructed on which the external observer recorded tutor performance and his/her activities as well as those of the group.

**Table 3:** Questionnaire

Dimension	Item no.	Item
Group interaction		
	1	Students posed adequate questions to each other to understand the learning content (e.g., questions on meaning of concepts, differences, reasons, and concrete examples)
	2	Group member statements were checked by asking each other critical questions.
	3	A group member who was formulating an explanation concerning the problem asked in between whether his/her explanation was right.
	4	One explanation did not suffice for the group members; alternative explanations were also given.
	5	Group members elaborated on each other’s arguments.
	6	When someone argued something, then that statement was challenged.
	7	Explanations of group members were amended with explanations of other group members.
	8	Students drew conclusions from the information that was discussed in the group.
	9	In the group, some contradictory beliefs on information concerning the learning content were present.
	10	One or more group members was/were contradicted by the others.
	11	When someone contradicted a group member, that person stated a counter-argument.
Effectiveness of tutors		The tutor encouraged us…
	12	… to summarize in our own words what we had learnt.
	13	… to search for links between issues discussed in the tutorial group.
	14	… to understand underlying mechanisms/theories.
	15	… to generate clear learning objectives on our own.
	16	… to search for various resources on our own.
	17	… to apply knowledge to the discussed problem.
	18	… to apply knowledge to other situations/problems.
	19	… to give constructive feedback on our group work.
	20	… to regularly evaluate group cooperation.
	21	The tutor had a clear view about his/her strengths and weakness as a tutor.
	22	The tutor was clearly motivated to fulfil his/her role as a tutor.
	23	Give a grade (1–10) for the overall performance of the tutor (6 being sufficient, 10 being excellent).
General comments on PBL		
	24	PBL suits my style of working.
	25	There was a pleasant working atmosphere in our small group.
	26	Overall, I am satisfied with the way our group handled comments and ideas.
	27	I was able to discuss issues that were important to me with the group members.
	28	I dealt with relevant topics during PBL sessions.
	29	I had the impression that I could practice “clinical thinking“when dealing with the cases.
	30	Summarizing my findings from the self-study session (in PBL step 7) provided useful information on my learning progress.
	31	The presented cases encouraged me to engage in self-study.
	32	The learning objectives of the group encouraged me to engage in self-study.
	33	I enjoyed the PBL sessions.
	34	I find it useful that students assume responsibility for the moderation.
	35	I welcomed the opportunity to moderate cases myself.
	36	Moderation by the other students stimulated my own contribution.
	37	The PBL tutor supported me in the role of moderator.
	38	Discussion of cases was a useful addition to the endodontics lectures.
	39	I consider the work with clinical cases in PBL sessions a useful part of my dental training.
	40	The range of discussed cases broadened my knowledge of endodontics.
	41	Case presentations by others were helpful to me. It was easy for me to participate in the discussion because…
	42	…I could readily picture the patients being discussed.
	43	…I had seen similar patients in the 9th semester when assisting in courses. I felt supported by my tutor with respect to my needs, expectations, views, etc.…
	44	…as a group member.
	45	…as moderator.
	46	…when I presented a case. The PBL tutor…
	47	…makes sure that the group defines problem issues.
	48	…activates my previous knowledge.
	49	…encourages me to contribute.
	50	…responds to contributions by team members.
	51	…assists me in putting things into context.
	52	…makes sure that the group formulates clear learning objectives.
	53	…makes sure that the learning objectives are discussed.
	54	…prevents digression from the topic.
	55	…assists me in visualizing results. The PBL tutor…
	56	…encourages group work.
	57	…describes well the way we work together.
	58	…helps the group to solve conflicts.
	59	I feel that the tutor was too strict (scores 1–2), just right (scores 3–5), or too lenient (scores 6–7).
	60	I feel that the PBL tutor has talked too much (scores 1–2), has talked exactly as much as needed (scores 3–5), or has not talked enough (scores 6–7).

### Qualitative part (Part 2)

The participating tutors were questioned about their experience with PBL by means of expert interviews that were half-standardized (semi-structured), i.e., the interviews were conducted using a guideline that only provided subject-related questions (Table 4).

**Table 4:** Guideline for expert interviews (*n* = 11 items)

Item no.	Item
1	How did your facilitative (f) or non-facilitative (nf) tutoring style affect the group discussion? Please describe a typical situation.
2	Did you observe a difference in the working atmosphere, depending on the tutoring style (f/nf) you applied?
3	Please describe in your own words how you perceived the differences between the tutoring styles (f/nf).
4	Which measures did you take to encourage the teamwork when applying the tutoring styles (f/nf)? How successful were you in your view?
5	Why do you think students can benefit particularly from PBL? Do you see any differences between the two tutoring styles (f/nf) in this respect?
6	How would you estimate your preparation effort for PBL? Do you feel that there are any differences in the expenditure of work between the two tutoring styles?
7	Which tutoring role made it easier for you to support the group with respect to the PBL procedure? Please explain.
8	How did you react to differences of opinion in the group, depending on the tutoring style you used (f/nf)?
9	What was your experience with the moderation of PBL cases by the students? Did you detect any differences, depending on the type of tutoring you assumed?
10	How did you feel in the role as a facilitative or non-facilitative tutor? Which role made it easier for you to perform the task of tutoring?
11	Do you think a PBL tutor should assume a facilitative or non-facilitative role?

One PBL group consisting of randomly allocated participants (*n* = 8 students) was exposed to both tutoring styles (facilitative or non-facilitative) in several sessions. These students participated in a focused group discussion to share their view about the two tutoring styles (Table 5). The setting of an open discussion allowed each participant to freely express his/her personal view and impression.

**Table 5:** Guideline for focus group discussion (*n* = 9 items)

Item no.	Item
1	How would you describe the performance of the tutor in the group?
2	In what way has the tutor encouraged the teamwork within the group? How successful was he/she in your view?
3	Did you benefit from PBL with respect to your studies? If yes, in what way?
4	How do you rate the preparation effort for PBL compared to other learning techniques?
5	In which situations did you feel insufficiently well supported by your tutor during the PBL sessions?
6	Were there any differences of opinion among the group members? If yes, how did the tutor react in such situations?
7	How did you cope with the fact that PBL cases were moderated by students? What did you consider useful? What was poor?
8	In summary, what aspects of PBL did you consider favorable?
9	In what way could PBL be further improved in your opinion?

The guidelines for expert interviews and the focused group discussion were prepared in collaboration with the Institute of Psychology, Department for Educational Psychology of the Goethe University of Frankfurt am Main. All interviews and the focused group discussion were recorded in both audio and video forms, using a HDC-SD40EG-K Full HD Camcorder (Panasonic K. K., Kadoma, Japan).

### Study evaluation

The quantitative data (Part 1) were evaluated in line with the intention-to-treat (ITT) principle. The following analyses were conducted:

1. quality criteria of the multiple-choice tests
2. analysis of study questionnaire, determination of reliability values and factors
3. explorative data analysis
4. mixed-model analysis (analysis of variance), taking group and tutor effects into account (significance level *p* ≤ 0.05).

All data were statistically analyzed using the software SAS 9.1 (SAS Institute Cary, NC, USA).

In Part 2 of the study (qualitative data), the recorded expert interviews and focused group discussion were transcribed, and relevant information was grouped by topic. The codes generated in this way were processed with the program MAXQDA (Verbi GmbH, Marburg, Germany) and analyzed in terms of a “thematic analysis” [37]. Two independent individuals who had been trained beforehand established the topic categories. These were based on interpretation of terms in the context in which they had been mentioned.

## Results

### Quantitative part (Part 1)

#### Dropout rate

The mean response rate for the total study population was 95.04 %, with a mean dropout rate amounting to 4.96 %.

### Activities of tutors and groups as noted by the external observers

Table 6 lists the findings with respect to tutor and group activities as perceived by the external observers.

**Table 6:** Tutor and group activities in groups tutored facilitatively (f) and non-facilitatively (nf). Data are means ± SD (SD = standard deviation)

Parameter	Facilitative (f)	Non-facilitative (nf)	Signifikance f/nf
Tutor activity	40.00 ± 24.17	26.96 ± 22.08	yes (*p* = 0.0047)
Group activity	124.57 ± 82.12	111.21 ± 38.99	no (*p* = 0.85)

### Factor analysis

Determination of the proper values for questions 1 to 60 in the explorative analysis of the questionnaire proved a 3 or 8-factor solution to be appropriate. The 8-factor approach allows a more detailed analysis; thus it was used for subsequent analyses. Table 7 shows the reliability estimates (Cronbach’s alpha), mean data, and *p*-values for the analyzed factors with the corresponding items of the questionnaire. Support by the tutor, tutor effectiveness, and motivation were significantly better in groups receiving facilitative tutoring than those receiving non-facilitative tutoring.

**Table 7:** Factors and corresponding items of the questionnaire (Values are Cronbach’s alpha (CA) and means ± SD (SD = standard deviation; plus *p*-values) for the 8-factor solution for groups supervised facilitatively (f) and non-facilitatively (nf)

Factor	Items	CA	Facilitative (f)	Non-facilitative (nf)	Signifikance f/nf
**Support**	19,20,44,47,51–58	0.87	4.14 ± 0.45	3.89 ± 0.43	**Yes (** ***p*** **= 0.003)**
**Tutor effectiveness**	12–18,21,22,45,46	0.85	4.05 ± 0.50	3.75 ± 0.49	**Yes (** ***p*** **< 0.001)**
Group interaction	1–8,25–27,42	0.81	3.96 ± 0.41	3.86 ± 0.39	No (*p* = 0.229)
Acceptabiliy	29–32,38–41	0.81	4.12 ± 0.56	4.04 ± 0.51	No (*p* = 0.54)
**Motivation**	24,28,33–35	0.75	4.01 ± 0.66	3.85 ± 0.46	**Yes (** ***p*** **= 0.04)**
Tutors’ overall performance	23,48–50,59,60	0.30	4.10 ± 0.38	4.02 ± 0.33	No (*p* = 0.09)
Moderation	36,37,43	0.53	3.32 ± 0.69	3.16 ± 0.68	No (*p* = 0.09)
Conflict potential	9–11	0.56	3.32 ± 0.66	3.26 ± 0.66	No (*p* = 0.6)

Significant differences are highlighted in bold

The dimension “tutor effectiveness” consisting of 5 subdimensions achieved mean (± SD) values ranging from 3.77 ± 0.82 (context-relevant learning) to 4.05 ± 0.63 (intrapersonal behaviour). For facilitative tutoring, the subdimensions reached the following mean scores: constructive, active learning: 4.08 ± 0.76 (versus 3.77 ± 0.85 for non-facilitative tutoring); self-driven learning: 4.22 ± 0.78 (versus 3.83 ± 0.92); context-relevant learning: 3.92 ± 0.79 (versus 3.62 ± 0.82); collaborative learning: 4.15 ± 0.78 (versus 3.87 ± 0.80); and intrapersonal behavior: 4.15 ± 0.80 (versus 3.96 ± 0.84).

### Statistical analysis of pre- and post-PBL tests

The overall results of the post-PBL test were significantly better than those of the pre-PBL test. The students reached a mean score of 16.05 ± 4.49 points in the pre-PBL test, and a mean of 21.38 ± 4.69 points computed for the post-PBL test (*p* ≤ 0.0001). After the pre-PBL test the students were stratified into the facilitative (with a mean of 16.30 points) and non-facilitative group (with a mean of 15.82 points) with no statistically significant difference (*p* = 0.590) between them.

In the post-PBL test both groups received significantly more correct answers than the respective pre-PBL group (*p* ≤ 0.05). Comparing the post-PBL results in the two tutoring styles (nf = 22.2 correct answers, f = 20.5 correct answers) no significant difference could be observed (*p* = 0.08).

### Qualitative part (Part 2)

#### Expert interviews

Table 8 shows the outcome (frequency of responses) of the focused group discussion with students and expert interviews with tutors. Additionally most tutors indicated that non-facilitative tutoring was difficult to apply (*n* = 7) and that previous training was necessary (*n* = 7). Irrespective of the tutoring style in their group, the students expressed uncertainty about the PBL procedure (*n* = 14). Moreover, the group composition was named to significantly influence the procedure (*n* = 8), and marked focusing on the tutor was observed (*n* = 2).

**Table 8:** Outcome of focused group discussion and expert interviews (with corresponding numbers indicating the frequency of responses)

	Facilitative tutoring (f)	Non-facilitative tutoring (nf)
Focus group discussion with students	1. Positive confirmation for students (7)	1. Uncertainess about pbl-process (10)
	2. Improved learning (4)	2. More effort (1)
	3. Higher degree of group interaction (3)	3. Lower degree of group interaction (3)
	4. Positive evaluation for facilitative behavior (20)	4. Negative evaluation for non-facilitative behavior (3)
Semi-structured interviews with tutors (group interaction)	1. Lower degree of students autonomy (2)	1. Students autonomy increases up to the second PBL meeting (6)
		2. High degree of students autonomy (5)
		3. Students’ need for support (1)
		4. Good cooperation (1) and high motivation (1)
Semi-structured interviews with tutors (tutor role)	1. Less stressfull for tutor (2)	1. High challenge for the tutor (5)
	2. Negative influence on learning (1)	2. Improved learning (3)
	3. Appropriate for PBL beginners (1)	3. Not appropriate for PBL beginners (5)

Similarly, the tutors considered PBL a challenging learning tool (*n* = 8) in which the group composition plays an important role (*n* = 3). They also concluded that PBL is easier to apply (*n* = 1) and requires less preparation time than classical teaching (*n* = 4). Suggestions for improving PBL included more practicing of the two different tutoring styles (*n* = 18), prolonged PBL meeting times (*n* = 3), and use of additional PBL cases (*n* = 4).

In the groups tutored non-facilitatively, tutors observed a high degree of student autonomy during the PBL sessions, particularly after the second session. They confirmed that “*students found out very many things on their own in the second session*“, and “*needed less eye contact*“. Furthermore, tutors concluded that the facilitative role is more suitable in a hybrid curriculum.

#### Focus group discussion

Table 8 shows the students‘assessment of tutoring styles and their effects on the PBL process. Additionally the students rated PBL as practice-oriented learning (*n* = 6) and favored the facilitative tutoring style (*n* = 20), with only one student rating the two tutoring styles as equally effective. Comments on the facilitative tutor were as follows: The facilitative tutor “tries to guide things into the right direction“; “is considered“, “does not use a poker face“, “was a supervisor and guide at the same time”; “provided feedback”; and “gave us an opportunity to ask questions”. In contrast, the non-facilitative tutor “was straight-faced“; tried to lead us astray“; and “solely acted as physician or patient”.

Suggestions for improving the module mostly concerned organizational issues (*n* = 10), followed in frequency by proposals of modifying the module contents (*n* = 6).

The students described the facilitative tutor role as a “*guideline that leads you along*“, and realized that “*it is not that bad if you say the wrong thing*“. In addition, they appreciated the fact that this tutoring style “*lets you know whether you go off in the wrong direction*”. The primary effect of non-facilitative tutoring on the group was uncertainty with respect to the PBL procedure. The students named the more practical mode of learning in PBL modules as the main advantage of PBL over conventional learning. Practice-oriented application of theoretical topics were considered “*good training for work with the patient*“; “*attempts to solve problems in a practical way*”, and a “*stimulating exercise*”. In the clinical sense, PBL was regarded as “*more effective*“, and “*the awareness for practical problems is being triggered*”. Students stated that for the first PBL cases, they would wish to have a facilitative tutor who subsequently “*becomes increasingly non-facilitative*”.

## Discussion

The tutor influences the success of PBL-groups. However the best way to perform the tutor’s role is a matter of debate. We compared two different tutor-styles. The facilitating tutor was more active, intervened and guided the group. The non-facilitating tutor only intervened in certain situations and kept still most of the time (see Tables 2 and 3).

There is general agreement in the available literature that PBL strengthens the learning motivation [2, 3, 6, 9]. In his flow theory, Csikszentmihalyi stated that “the structure of activity in the context of challenge, goal, feedback, concentration, and control has major influences on intrinsic motivation [38, 39]. “Flow” is defined as “the holistic experience that people feel when they act with total involvement”. In line with these statements, our study revealed a significant difference in the variable “motivation“between the two tutoring styles in favor of facilitative tutoring (4.01 ± 0.66 versus 3.85 ± 0.46).

The style of PBL tutoring affects the group work in a direct manner. For the present study, we used some of the dimensions published by Dolmans & Ginns [35]. In line with the PBL form used at Charité, Berlin, our questionnaire consisted of 60 items. Some items (items 47–55) were phrased in a way that may have favored the facilitative style, which should be critically stated. The highest values in the rating of tutors occurred in the subdimension “intrapersonal behavior”. Facilitative tutors were more effective in “generating clear learning objectives“encouraging “the search for additional sources of information“, which was reflected in the group members’ assessment of tutors. To optimize group work, Wood [40] suggested allocating specific roles to the group members before sessions (e.g., reader, writer, time keeper, moderator, and summarist). We used these roles also in the present study.

PBL evaluation consists of assessing the students as well as the program [14, 41]. Such evaluations are difficult to conduct, and several differing approaches have been described in the literature. By employing multiple-choice tests, Budé et al. [42] demonstrated a significant difference (*p* = 0.072) between so-called directive and traditional PBL modules, with the directive programs achieving a better result. In our study, knowledge gain in the post-PBL test was significant in both groups, reflecting the newly acquired knowledge after completing the PBL module. There was a trend towards better results in the group tutored non-facilitatively, but the difference did not reach statistical significance.

In the expert interviews, tutors expressed clear difficulties with their non-facilitative role. Moreover, tutors documented more problems in implementing a non-facilitative tutoring style with PBL beginners, as apparent by the following comments: “*My personal experience is that the non-facilitative role is not easy for a student who has never participated in a PBL module*“; “*If group members have already undergone a PBL session, I feel comfortable in either role*”; or “*The first session was really difficult when assuming the non-facilitative role*”. The comment “*The differences between the tutor roles and their practical implementation were not entirely clear in the role description*“suggests that there was a need for more extensive tutor training. In line with this observation, the need for more extensive tutor training is emphasized in the literature [43–46].

With respect to internal study validity, the aspects to be considered include maturity, selection of study participants, study location, instrumentation, and the special study design used. During the 8-week PBL module „Endodontics“, students attended multiple other lectures and courses that were not directly connected with the topic of the PBL module but may have indirectly affected the monitoring of learning objectives. To minimize this effect, all programs on similar topics organized by the outpatient clinic for restorative dentistry were scheduled to take place after completion of the PBL module. Maturation’ may possibly also have affected the tutors involved in the study. It cannot be ruled out that the tutors became increasingly accustomed to their specific role during the study. This factor was controlled by the external observers who confirmed the assigned roles. To reduce potential problems associated with the study location, all PBL sessions were conducted in the same room (simulation laboratory) and took place on the same week day (Tuesday) at the same time (2 p.m. to 5 p.m.).

The issue of instrumentation concerned the two written tests that were conducted before and after completion of the PBL module. Since these had to remain the same throughout the three semesters, the questions and answers were not published or discussed at any time. For each semester, the test documentation contained identical questions, but they were presented in a different order than those of the previous semester. All tests were conducted without announcing them; neither the students nor the tutors knew the exact date of the tests.

The four tutors taking part in the study were responsible for the implementation of the different roles during the PBL sessions. All of them had many years of experience with PBL. However, it was feared that some of them might leave the university before the end of the study because of an expiring contract, which constituted a substantial complication in the planning of the study. The chosen study design (“nested“design) appeared to provide the best condition for this potential complication in that it allowed for a maximum size of factor variance. For this reason, the tutors had to switch roles each time, and the various PBL cases were exchanged during the module. In this way, replacing a tutor would have been less critical.

The chosen study design also addressed the potential problem of implementation, i.e., the specific selection of a role for each tutor and free allocation of students to a tutorial. Any interaction between groups (tutored by a facilitative or non-facilitative style) was minimized by the simultaneous scheduling of PBL-sessions with only the same tutoring style. Thus, potential variation in tutor performance was not evident in the parallel sessions. Any confounding by student interaction outside the PBL module was kept to a minimum by blinding the participants with respect to the study variables.

Our study design consisting of pre- and post-PBL assessments may be associated with certain limitations in connection with the testing condition [47]. There is a danger that students anticipate the tests and will therefore not be unprepared when attending PBL sessions. Moreover, based on their experience with the nature of information and type of questions in the pre-PBL test, participants can prepare more effectively for the post-PBL test than for the pre-PBL test. Nonetheless, Fraenkel & Wallen [47] also point to the advantages of this study design; it provides information on the group composition with respect to the knowledge level and consequently on the comparability of the two groups. The authors point out that this study design is particularly useful if groups contain no more than 30 participants. With as many as 101 participants in our study, this limit was clearly exceeded. The large group size in our study resulted from the combination of three semesters. Consequently, pre-PBL testing was of critical importance to ensure comparable knowledge levels among the groups. In our study, the groups were, in fact, comparable with respect to their current knowledge on the subject.

An additional aspect of debate is the number of study participants. Within the study time frame, it was not possible to recruit more students (total number of students per semester = 48) and tutors (total number of experienced PBL tutors available at the entire dental school = 5) at our institute. Sample-size calculation prior to study start yielded a number of at least 50 participants per group (facilitative versus non-facilitative), but this number could not even have been met by adding students from an additional semester. Nonetheless, it would be desirable to confirm our findings in subsequent studies involving larger study populations.

With respect to external validity of the study, generalizability of our findings and the special setting of a replication study are of interest. To ensure that our results can be transferred to other persons, situations, conditions, and time frames, we allocated students to one of two groups by stratified randomization. Moreover, the low dropout rate increased the external validity of this study. Further strengthening of the external validity resulted from the nature of our evaluation that can be viewed as a replication study. Because the study ran over a period of three semesters, findings were transferable to other study subjects as different students were involved.

The “readiness” of tutors and students underwent a certain process. Initially, the facilitative style was favored by both the tutors and students. In the Results section we state that the facilitative tutoring style had resulted in less self-directed learning by the end of the PBL experiments. The tutors mentioned that the non-facilitative tutoring style greatly increased the students’ ability to learn on their own.

## Conclusions

Within the limitations of the study that was based on a hybrid curriculum, we conclude that the tutor style should be modified during PBL training. Initially, the facilitative style may be more suitable, but the non-facilitative tutoring style should be introduced when students have gained sufficient knowledge in PBL. We believe that this approach would be of optimal long-term benefit to both students and tutors participating in PBL programs.
